# Mechanistic insights curcumin’s anti-inflammatory in pancreatic cancer: experimental and computational evidence implicating IL1B interference *via* IL10RA upregulation and NLRP3/TLR3 downregulation

**DOI:** 10.3389/fcell.2025.1601908

**Published:** 2025-06-04

**Authors:** Jun-Feng Cao, Kuan Hang, Hao Zhang, Qingjie Xia, Xiao Zhang, Jie Men, Jin Tian, Zengliang Xia, Dunshui Liao, Kezhou Li

**Affiliations:** ^1^ Division of Pancreatic Surgery, Department of General Surgery, West China Hospital, Sichuan University, Chengdu, Sichuan, China; ^2^ College of Medicine, Southwest Jiaotong University, Chengdu, Sichuan, China; ^3^ Institute of Neurological Diseases, Translation Neuroscience Center, West China Hospital, Sichuan University, Chengdu, Sichuan, China; ^4^ Chengdu Medical College, Chengdu, Sichuan, China

**Keywords:** curcumin, pancreatic cancer, machine learning, transcriptome sequencing, cellular experiments, computer simulation

## Abstract

**Purpose:**

Pancreatic cancer is a highly aggressive malignancy characterised by a complex tumour microenvironment and chronic inflammation. Studies found curcumin inhibited with inflammatory responses and tumour proliferation by interfering with production and activation of pro-inflammatory factors. This study investigated curcumin treated pancreatic cancer by modulating key targets in the inflammatory response and their signalling pathways.

**Methods:**

The human pancreatic cancer PL45 cells and SUIT-2 cells were utilized to establish cellular experiments, and the effects of curcumin on proliferation, apoptosis and cell migration of PL45 cells and SUIT-2 cells were detected by CCK-8, Annexin V-FITC/PI and cell scratching experiment. PL45 cells RNA from experimental and control groups was also analyzed by transcriptome sequencing. Bioinformatics screening of differential gene targets in transcriptome sequencing was performed. Gene Ontology, KEGG and Protein-protein interaction were used to analyze the differentially expressed targets at the gene level and protein level, respectively. We validated the differential gene targets by machine learning analysis of GSE28735 data, and performed survival analysis, pan-tumor analysis, immune infiltration analysis and single-cell transcriptional analysis on the differentially expressed targets. Computer simulations were utilized to verify the stability of curcumin binding to key proteins.

**Results:**

Results of cellular experiments suggested 30 μg/mL curcumin and 50 μg/mL curcumin significantly inhibited the proliferation and growth of PL45 and SUIT-2, respectively. The transcriptome results indicated that 2,676 genes showed differential expression in curcumin-treated group compared to control group. Bioinformatics and machine learning analyses screened 14 key targets that are closely related to the inflammatory response in pancreatic cancer. Molecular dynamics showed binding free energies for IL1B/Curcumin, IL10RA/Curcumin, NLRP3/Curcumin and TLR3/Curcumin were −12.76 ± 1.41 kcal/mol, −11.42 ± 2.57 kcal/mol, −28.16 ± 3.11 kcal/mol and −12.54 ± 4.80 kcal/mol, respectively.

**Conclusion:**

This research findings indicated that curcumin not only directly interfered with the activation of IL1B through blocking activation of NLRP3 by TLR3, but also upregulated expression of IL10RA to activate IL-10, thereby interfering with IL1B and its downstream signalling pathway.

## Graphical Abstract

**Figure:**
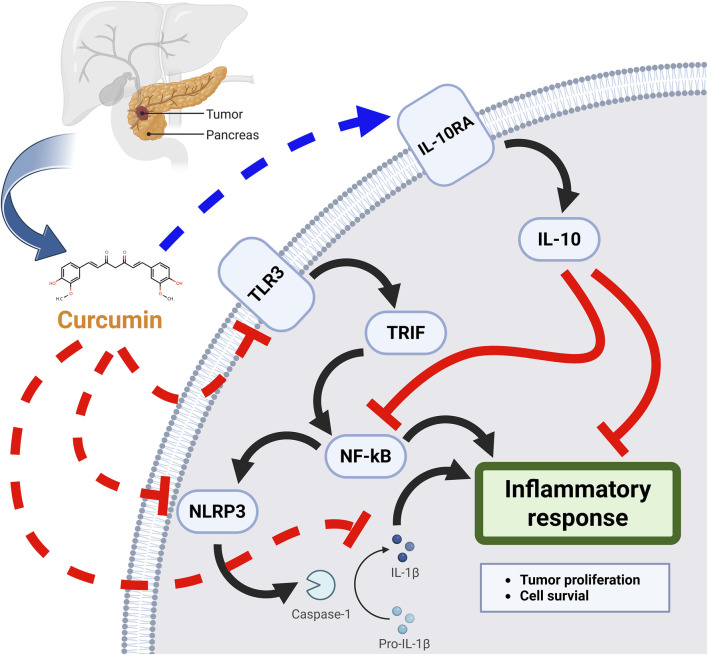
The mechanisms of curcumin inhibits inflammatory response thereby treating pancreatic cancer.

## Introduction

Pancreatic cancer was an aggressive malignant tumor, the biological behavior of pancreatic cancer was regulated by multiple cellular processes, including inflammatory responses and immune evasion (Mayerle et al., 2019; Poulia et al., 2020). Study has increasingly focused on the critical role of inflammatory responses in the onset and progression of pancreatic cancer. Inflammation is not only a significant risk factor for pancreatic cancer but also influences biological behavior of cancer through various signaling pathways. The interactions and regulations among these signaling pathways form a complex network that profoundly affects cancer progression (Caronni et al., 2023; Ye et al., 2020).

NF-κB pathway is one of central regulators of inflammatory response (Shostak and Chariot, 2015). NF-κB promotes tumor proliferation and cell survival by regulating release of pro-inflammatory factors while inhibiting apoptosis (Mirzaei et al., 2022; Thomas-Jardin et al., 2020). NF-κB pathway can also interact with JAK/STAT3 to further enhance proliferation and anti-apoptotic capabilities of tumor (Shiraiwa et al., 2019). Additionally, COX-2/PGE2 signaling pathway enhances inflammatory response in tumor microenvironment and promotes angiogenesis by synthesizing prostaglandins (Ali et al., 2022; Gray et al., 2014; Yu et al., 2020). TGF-β pathway has dual roles; it can inhibit early tumor growth and promote tumor invasion and metastasis in later stages (Renaldi et al., 2021; Derynck and Budi, 2019; Zhai et al., 2022). TGF-β can influence behavior of tumor by modulating NF-κB and STAT3 (Carvalho et al., 2023). Furthermore, these signaling pathways can regulate immune evasion capabilities of tumors through regulating immune checkpoint molecules (Gough et al., 2021).

Interleukin family played a crucial role in inhibiting inflammatory responses, which significantly impacted treatment of pancreatic cancer and holds potential clinical value (Shadhu and Xi, 2019; Holmer et al., 2014). And interleukin family influences the biological behavior and life processes of pancreatic cancer by several signaling pathways (Li et al., 2023a). IL6 is one of main activators of JAK/STAT3. By inhibiting IL6, it is possible to reduce the phosphorylation of STAT3, thereby decreasing the proliferation and anti-apoptotic capabilities of tumor (Kaur et al., 2020; Johnson et al., 2018). And inhibiting activity of IL6 can significantly affect activation of JAK/STAT3 signaling pathway. This intervention not only suppresses tumor growth but also promotes apoptosis of tumor by downregulating protein expression of cell cycle and upregulating protein expression of apoptosis (Li et al., 2023b; Chen et al., 2022a). Study found inhibiting IL6 could also reduce activity of NF-κB pathway, lower production of inflammatory mediators and mitigate the pro-inflammatory state within tumor microenvironment (Zhu et al., 2022). Furthermore, IL8 affects cancer migration and invasion by regulating activity of MAPK and NF-κB (Lian et al., 2022; Song et al., 2020; Song et al., 2018). IL8 in tumor microenvironment enhances invasiveness and motility of tumor by inducing EMT (Fousek et al., 2021). Research found inhibiting IL8 could prevent occurrence of EMT, reduce cytoskeletal reorganization, and decrease the expression of cell adhesion molecules, thereby reducing tumor metastasis (Matsushima et al., 2022). Additionally, inhibiting IL8 can also reduce angiogenesis, limiting the tumor’s supply of oxygen and nutrients, further inhibiting tumor spread and growth (Alraouji and Aboussekhra, 2021; Alfaro et al., 2017). IL-10, as an immunosuppressive interleukin, reduces tissue damage by inhibiting inflammatory responses (Rallis et al., 2022). Some studies suggested that inhibiting IL1B activity could decrease activity of MAPK and NF-κB pathways, thereby reducing inflammatory responses and tumor cell proliferation. And IL1B promoted tumor invasion and growth by inducing production of various pro-inflammatory factors and enhancing inflammatory responses (Kang et al., 2021; Xia et al., 2018). By inhibiting IL1B, production of inflammatory mediators can be reduced, weakening the pro-inflammatory state in tumor microenvironment, thereby inhibiting tumor progression (Pretre et al., 2022). NLRP3 participates in recognizing cellular danger signals, triggering a series of downstream reactions by sensing changes in external and internal environment of cells (Sharma and Kanneganti, 2021; Tengesdal et al., 2023). Upstream signal proteins of NLRP3 include signal pathways mediated by receptors such as TLR and NOD, and these proteins promote transcription and promoter activation of NLRP3 by stimulating NF-κB and MAPK pathways (Tengesdal et al., 2023; Unterberger et al., 2021; Xu and Núñez, 2023). CASP1 was not only responsible for release and maturation of pro-inflammatory cytokines, but it also initiated pyroptosis, a type of programmed cell death that further amplifies the inflammatory response and influences cancer progression (Fu and Wu, 2023; Makoni and Nichols, 2021; Wang X. et al., 2023; Yang et al., 2023a). CSF2 not only promoted differentiation and proliferation of monocytes and macrophages but also played an essential role in regulating tumor microenvironment (Xu et al., 2019; Patel et al., 2020; Kumari et al., 2019).

Curcumin is a polyphenolic compound found primarily in rhizomes of turmeric (Huang et al., 2022; Zhao et al., 2023; Kotha and Luthria, 2019). The chemical structure of curcumin consists of two aromatic rings connected by a seven-carbon linker, with multiple phenolic hydroxyl groups and conjugated double bonds that confer unique biological activities (Nelson et al., 2017; Hatamipour et al., 2018). The low toxicity of curcumin and its analogs and the synergistic sensitizing effect with standard chemotherapy confer an important adjuvant status and development prospect in the comprehensive treatment of pancreatic cancer (Nagaraju et al., 2019; Tomeh et al., 2019).

Research indicated that curcumin could downregulate gene expression of pro-inflammatory factors in the interleukin family while inhibiting growth of pancreatic cancer (Mollazadeh et al., 2019; Ghandadi and Sahebkar, 2017; Hasanzadeh et al., 2020). This inhibitory effect led to a marked decrease in gene expression of pro-inflammatory factors including IL6, IL8 and IL1B, reducing the pro-inflammatory state within tumor microenvironment (Marquardt et al., 2015; Aggarwal et al., 2006). Curcumin suppressed inflammatory response of pancreatic cancer by inhibiting pro-inflammatory factors, thereby reducing the support that inflammatory mediators provide for tumor cell survival and proliferation. Curcumin reduced phosphorylation of STAT3 by downregulating IL6 expression, thereby diminishing the proliferation and anti-apoptotic capabilities of tumor (Li et al., 2020; Zhang et al., 2019; Afshari et al., 2023; Khan et al., 2020). Curcumin also interfered with the epithelial-mesenchymal transition by regulating TGF-β, consequently reducing invasive and migration abilities of tumor (Kandagalla et al., 2022; Datta et al., 2013). Curcumin exerted its anticancer effect by regulating activity of NLRP3 inflammasome (Du et al., 2023; Joshi et al., 2021). Curcumin might reduce inflammatory response intensity by inhibiting activity of CASP1 and decreasing release of pro-inflammatory cytokines (Li et al., 2004; Siwak et al., 2005). And curcumin inhibited inflammation-related cancer cell proliferation by reducing IL1B maturation and release through inhibition of CASP1 activation. (Miller et al., 2014; Jaiswal et al., 2022; Gong et al., 2015). Curcumin reduced the occurrence of DNA damage and mutations by scavenging free radicals, thereby inhibiting tumor development (Joshi et al., 2023; Yin et al., 2018). Curcumin effectively attenuated the inflammatory microenvironment of pancreatic cancer by modulating a complex signaling network that downregulates NLRP3, CASP1 and CSF2 while simultaneously regulating expression of key pro-inflammatory factors within interleukin family. This multi-target regulatory mechanism not only shows curcumin’s strong potential in tumor prevention and treatment, but also provides an essential scientific basis for further development of more precise cancer therapies.

The resultant studies showed that both curcumin and its analogues were effective in delaying pancreatic tumor progression, but their low bioavailability and rapid metabolism in humans limited the expected clinical benefit (Unlu et al., 2016). Moreover, the specific mechanism of curcumin and its analogs in the treatment of pancreatic cancer is still unclear (Wang Wei et al., 2023). Conducting studies related to curcumin treatment of tumors will further promote the combination of curcumin and its analogs with molecular targeting agents, immunotherapy and other new strategies, thereby improving the clinical prognosis and quality of survival of pancreatic cancer patients.

## Material and methods

### Cellular experiment

The pancreatic ductal adenocarcinoma PL45 cell and the pancreatic cancer SUIT-2 cell used in cellular experiment were purchased from Nanjing Kebai Biological company and Ubigene company. High-glucose DMEM used to culture PL45 cells and SUIT-2 cell was obtained from Hyclone, USA. The culture medium contained 10% FBS purchased from Gibco, USA, and 1% penicillin-streptomycin obtained from Sigma, USA. Following cell resuscitation, they were seeded in 25 mm^2^ culture flasks and incubated at 37°C in an incubator with 5% CO_2_. Cells were passaged when reaching a density of 75%–85% (Zhong et al., 2020; Cao et al., 2025). After stable passaging through three generations, PL45 and SUIT-2 were plated into 96-well plates, with each well containing 200 μL of medium at a concentration of 1 × 10^5^ cells (Akl et al., 2022).

High-purity curcumin was purchased from Macklin company. Curcumin was dissolved in DMSO and ultrasonically agitated before being used in cell experiments. In these cell experiments, a control group received 0.1% DMSO (without curcumin), and treatment groups were exposed to curcumin at 5, 10, 20, 30, 40, 50, 60, 80 and 100 μg/mL. To ensure reliable data, each group was established in six replicates. PL45 cells and SUIT-2 cells in 96-well plates were cultured in complete medium for 36 h before curcumin was added to allow for adherent growth of cells. Throughout the experiment, all groups underwent a 24-h treatment with curcumin. Subsequently, culture medium in each well of 96-well plate was discarded, 200 μL high-glucose DMEM culture medium containing 10% CCK-8 was added to each well (Yang et al., 2023b; Tang et al., 2021). After 4 h incubation in incubator, the absorbance of each well was measured at 450 nm using enzyme marker to assess impact of curcumin at different concentrations on proliferation of PL45 cells and SUIT-2 cells (Shi et al., 2023). In the cell scratch assay, PL45 cells and SUIT-2 cells inoculated in 6-well plates were grown to more than 80% confluence, 200 μL sterile pipette tip was used to scratch on the cell monolayer in the same direction, and after gentle rinsing to remove cellular debris, curcumin-containing or drug-free medium was added to continue the incubation, and the healing of the scratches was recorded by taking pictures with an inverted microscope after 24 h of incubation in the incubator. Apoptosis was detected using Annexin V-FITC/PI kit (Yang et al., 2024). Cells in the control and curcumin-treated groups were washed with PBS, Annexin V-FITC probe and PI staining solution were added, incubated for 15 min away from light, and observed and photographed under a fluorescence microscope, which was used to qualitatively evaluate the effects of curcumin treatment on early and late apoptosis of pancreatic cancer cells.

### Transcriptome sequencing

After plating PL45 cells onto a 6-well plate, culture them until they reach a cell density of 80%–85%. Both control group and curcumin treatment group were set up with three replicate wells each, followed by drug treatment for 24 h. After drug treatment, the supernatant in the 6-well plate was discarded. And TRIzol reagent was used to isolate total RNA from the cells (Chen et al., 2022b). The extracted RNA samples were cleaned and filtered to obtain high-quality reads. This extracted RNA was evaluated for purity, concentration, and integrity to guarantee reliable downstream analyses. Bowtie two then aligned the reads to the reference genome, establishing a robust mapping framework. Subsequently, RSEM was employed to quantify gene expression levels across all samples (Gong et al., 2024). This study analyzes the regulation and impact of curcumin on gene expression in PL45 cells through transcriptome analysis.

### Gene target enrichment analysis

The differentially expressed genes identified as upregulated and downregulated by transcriptome sequencing were subjected to GO functional annotation and KEGG pathway enrichment analysis by DAVID. Python was used for preprocessing tasks including normalization and correction of the raw transcriptome data. The processed data was then imported into DAVID for screening and analysis (Ma et al., 2022; Cao et al., 2022a). GO functional annotation helped identify cellular component, molecular function and biological process associated with the gene targets of differential expression. KEGG identified related signaling pathways and main biological processes through which curcumin inhibited pancreatic cancer growth and inflammatory responses. Gene target screening was performed with a significance threshold of *p* < 0.05, aiming to pinpoint critical signaling pathways and biological processes relevant to pancreatic cancer.

### Protein-Protein interaction construction and screening key protein targets

This study focused on regulation and impact of curcumin on inflammatory response in pancreatic cancer. This study obtained relevant gene targets from Genecards using the keywords “pancreatic cancer” and “inflammatory response”. Furthermore, we identified intersecting gene targets by screening transcriptome sequencing results and gene targets related to pancreatic cancer and inflammatory response (Bai et al., 2024). Relevance score of inflammation response ≥10 was set as the threshold to identify potential gene targets for curcumin’s regulation of inflammation response in pancreatic cancer. STRING was used to analyze PPI network associated with curcumin’s regulation of inflammation in pancreatic cancer. Cytoscape 3.9.1 was employed to evaluate network topology parameters, and key protein targets were selected based on a node degree and average centrality exceeding their respective median values.

### Transcriptome data processing for clinical samples and machine learning

GSE28735 is a microarray gene expression profile of tumors and adjacent non-tumor tissues of pancreatic ductal adenocarcinoma patients obtained from the NCBI GEO database. In this study, 45 matched pairs of gene expression of pancreatic tumors and adjacent non-tumor tissues from 45 patients with pancreatic ductal adenocarcinoma were selected and log-transformed. Differentially expressed genes between adjacent non-tumor and pancreatic tumor tissue samples were analyzed and compared using “Limma” R software package. To identify potential targets of pancreatic cancer and inflammatory response, we used three machine learning methods: Random Forest (RF), Support Vector Machine Recursive Feature Elimination (SVM-RFE) and Least Absolute Shrinkage and Selection Operator (LASSO). Each method provided a unique perspective on feature selection and model development. The Random Forest approach improved model accuracy by building multiple decision trees and combining their predictions through voting or averaging. By introducing randomness, RF mitigated risk of overfitting typically associated with single decision trees, making it particularly effective for large datasets. RF not only handled high-dimensional data comfortably but also provided importance scores for features, allowing us to discern which biomarkers were most valuable for early diagnosis of pancreatic cancer. LASSO, a linear regression method, facilitated feature selection by applying an L1 penalty to regression coefficients, thereby effectively reducing model complexity and improving predictive performance. We utilized the glmnet package to implement 10-fold cross-validation, ensuring the stability and generalizability of the selected features across different data partitions. LASSO was particularly well-suited for high-dimensional data analysis, as it excelled in feature selection and can adeptly address multicollinearity issues. SVM-RFE was a feature selection technique based on support vector machines that efficiently filtered features by training the model and assessing feature importance. This method excelled at identifying relevant features and eliminating redundant ones, outperforming traditional linear discriminant analysis and mean squared error methods. By progressively removing less influential features, SVM-RFE sought to uncover the optimal feature set, thereby enhancing the generalization capabilities of the final model. The integrated application of LASSO, SVM-RFE and RF provided a complementary approach that enhances the comprehensiveness of our analysis. LASSO introduces an automated feature selection process, effectively controlling the complexity of the model. SVM-RFE refined the feature selection further, ensuring the representativeness of the genes. Meanwhile, RF offered robust support in preventing overfitting, thereby strengthening the overall performance of the model.

### Survival analysis, pan-tumor analysis, immune infiltration analysis and single-cell transcriptional analysis of key differentially expressed genes

To comprehensively examine the link between pivotal differentially expressed targets and clinical treatment outcomes, we retrieved pancreatic cancer patient survival data from GEPIA database. This enabled us to investigate correlation between patient survival and expression level of specific genes. Through this analysis, we identified critical differentially expressed genes associated with poor prognosis. Subsequently, we conducted a pan-cancer analysis of these key differentially expressed genes using Tumor Immune Estimation Resource (TIMER). TIMER focuses on immune cell infiltration within tumor microenvironment and provides relevant data on gene expression and immune cell infiltration across various cancer types. By performing a pan-cancer analysis of key differentially expressed genes, we were able to assess their prevalence and specificity across different tumor types. Moreover, immune infiltration analysis by CIBERSORT was performed in this study. The expression levels of the differential genes in relation to immune cell subpopulations in the tumor microenvironment were quantitatively analyzed to assess potential impact of these genes on inflammatory response *versus* tumor immune regulation in cancer tissues. We verified the expression and distribution of key differentially expressed genes in different cells of pancreatic cancer by single-cell sequencing results of pancreatic cancer from TISCH2 database, so as to analyze the impact of key differentially expressed genes on the tumor microenvironment of pancreatic cancer. TISCH2 is a scRNA-seq database focused on the tumor immune microenvironment. We performed single-cell sequencing analysis of key genes by CRA001160 (PMID: 31273297) and GSE154778 (PMID: 32988401) in TISCH2. CRA001160 and GSE154778 contain single-cell sequencing results from 35 to 16 pancreatic cancer patient samples, respectively.

### Molecular docking

To conduct a more comprehensive analysis of interrelationships among curcumin and inflammation-related proteins in pancreatic cancer, we used molecular docking for simulation studies. The protein structures and curcumin structure used for molecular docking were obtained from PDB database and PubChem database, respectively. Before molecular docking, all structures were subjected to energy minimization within the MMFF94 force field to ensure conformational stability (Rhabori et al., 2022; Cao et al., 2022b). We utilized AutoDock Vina version 1.2.3 for molecular docking. We used PyMol 2.5.5 to preprocess receptor proteins by removing salt ions, water molecule and other substances to prevent unnecessary interference during the docking process. ADFRsuite was involved in converting the processed curcumin and protein into PDBQT format required by AutoDock Vina. This step was essential to ensure that the docking software correctly recognizes and handles molecular structures (Siddiqui et al., 2021). The conformation with the highest docking score was deemed the optimal binding conformation and was subsequently used for molecular dynamics to confirm its stability and biological relevance.

### Molecular dynamics

Molecular dynamics was employed to further evaluate and analyze the dynamic changes following the binding of curcumin to protein target. During molecular dynamics simulation, we used the curcumin-protein complex obtained from molecular docking as initial structure. AMBER 22 was utilized for conducting molecular dynamics simulation. Prior to the simulation, curcumin and proteins were processed using GAFF2 small molecule force field and ff14SB protein force field to provide suitable force field parameters for simulating intermolecular interactions. LEaP module was utilized to add hydrogen atoms to each system to simulate a water environment under physiological conditions (Pandi et al., 2022; Cao et al., 2022c). Before molecular dynamics, energy optimization was performed to eliminate unreasonable contacts and stresses in the initial structure. After completing energy optimization, the system underwent 200 ps heating process at a constant volume and a constant heating rate, gradually raising temperature from 0 K to 298.15 K. Once the preparation steps were complete, 100 ns molecular dynamics was conducted on the composite system.

### Statistical analysis

In this study, the Student’s t-test was used to assess significance of differences between groups. Results were considered statistically significant at *p* < 0.05, indicating a significant difference between the data outcomes. Study data were reported as mean ± standard error of the mean (S.E.M.) to illustrate central tendency and variability.

## Results

### Curcumin significantly inhibits PL45 cells and SUIT-2 cells proliferation activity

**FIGURE 1:**
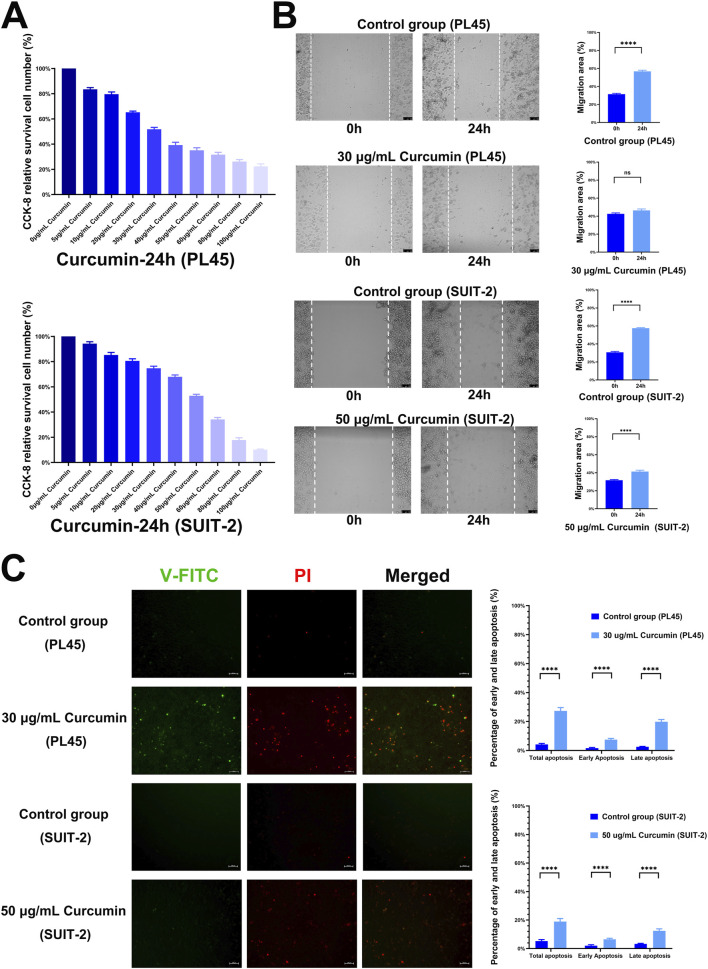
Results of cellular experiments. **(A)** The results of cell viability by Cell Counting Kit-8 (CCK-8) (n = 6), the effects of curcumin on cell proliferation were detected after treating PL45 and SUIT-2 cells with different concentrations of curcumin for 24 h, respectively, **(B)** Results of cell scratch assay (n = 8), after 24 h of treatment, 30 μg/mL curcumin and 50 μg/mL curcumin inhibited the migration of PL45 cells and SUIT-2 cells, respectively, **(C)** Results of Annexin V-FITC/PI cell apoptosis assay.

This study established pancreatic cancer cell models using PL45 cells and SUIT-2 cells. Results showed curcumin could significantly inhibit proliferation activity of PL45 and SUIT-2. Cellular experiments results indicated a concentration-dependent inhibitory effect of curcumin on pancreatic cancer cell proliferation. Notably, 30 μg/mL curcumin showed a distinctly strong suppression of PL45 proliferation, reaching IC50 under this treatment. And the inhibition of SUIT-2 proliferation by 50 μg/mL curcumin reached IC50 (Figure 1A). In the cell migration assay, the migration area of cells in control group was significantly increased within 24 h. And 30 μg/mL curcumin and 50 μg/mL curcumin significantly inhibited the cell migration of PL45 and SUIT-2, respectively. The results of cell migration assay showed curcumin could effectively inhibit migration ability of pancreatic cancer cell. This result suggested curcumin could reduce invasiveness of tumor by inhibiting motility of tumor cells (Figure 1B). Fluorescence apoptosis assay results showed proportion of early and late apoptotic cells in the curcumin-treated group was significantly higher than that in control group, indicating that curcumin could induce apoptosis in pancreatic cancer cells (Figure 1C). These results suggested that curcumin not only inhibited migration and proliferation of pancreatic cancer cell, but also enhanced its anti-tumor effect by inducing apoptosis.

### Transcriptomic analysis and GO and KEGG analysis

**FIGURE 2:**
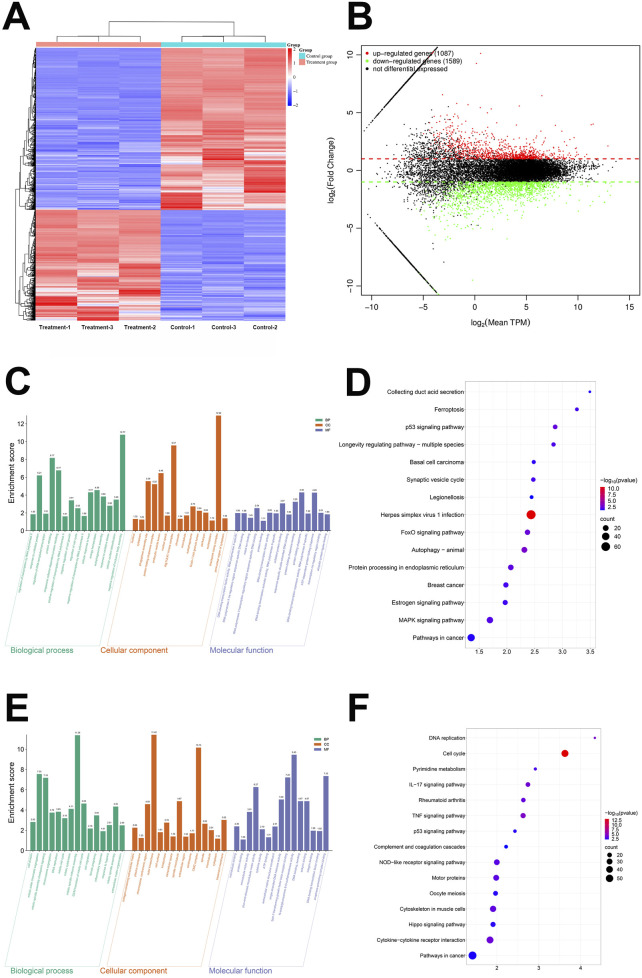
The results of transcriptome sequencing, Gene Ontology (GO) and Kyoto Encyclopedia of Genes and Genomes (KEGG) enrichment analysis. **(A)** The heat map of microarray (n = 3), **(B)** The Volcano plot of microarray (n = 3), **(C)** The GO enrichment analysis of upregulated gene, **(D)** The KEGG enrichment analysis of upregulated gene, **(E)** The GO enrichment analysis of downregulated gene, **(F)** The KEGG enrichment analysis of downregulated gene.

To further understand regulatory relationship of curcumin on PL45 pancreatic cancer cells at the genetic level, we performed transcriptome sequencing on groups treated with 30 μg/mL curcumin and control groups. The transcriptome results indicated that 2,676 genes showed differential expression in curcumin-treated group compared to control group. Among these, 1,589 genes were downregulated and 1,087 genes were upregulated (Figures 2A,B). To comprehensively analyze signaling pathway and biological process involved in differential expression of these genes, we conducted GO and KEGG analyses on 1,589 downregulated genes and 1,087 upregulated genes separately. In the upregulated differentially expressed genes, GO analysis identified 255 entries, including 159 BP, 38 CC and 58 MF. Biological processes were associated with cell proliferation and DNA replication, with particular involvement of regulation of cell cycle negative and regulation of cell growth. In terms of cellular components, nucleus, cytoplasm and protein folding chaperone complex were prominently featured. Regarding molecular functions, there was a higher presence of DNA-binding transcription factor activity (Figure 2C). KEGG analysis identified 18 entries. The signaling pathways primarily involved inflammatory response and apoptosis, including autophagy, FOXO signaling pathway, among others (Figure 2D). In the downregulated differentially expressed genes, GO analysis identified 551 entries, including 367 BP, 97 CC and 87 MF. Biological processes were associated with cell growth and inflammation, with particular involvement of cell division, mitotic cell cycle and inflammatory response. In terms of cellular components, plasma membrane and cell surface were prominently featured. Regarding molecular functions, there was a higher presence of microtubule binding, cytokine activity and ATP binding (Figure 2E). KEGG analysis identified 57 entries. The signaling pathways primarily involved cell growth and inflammatory damage, including cell cycle, tissue damage signaling pathway, among others (Figure 2F). The results revealed that curcumin synergistically upregulated apoptosis, stress and cycle inhibition-related genes, and downregulated proliferation, inflammation and tissue damage-related genes through multiple pathways, thereby inhibiting pancreatic cancer cell growth and invasion at multiple levels.

### Protein-Protein interaction analysis and screening of key protein targets

**FIGURE 3:**
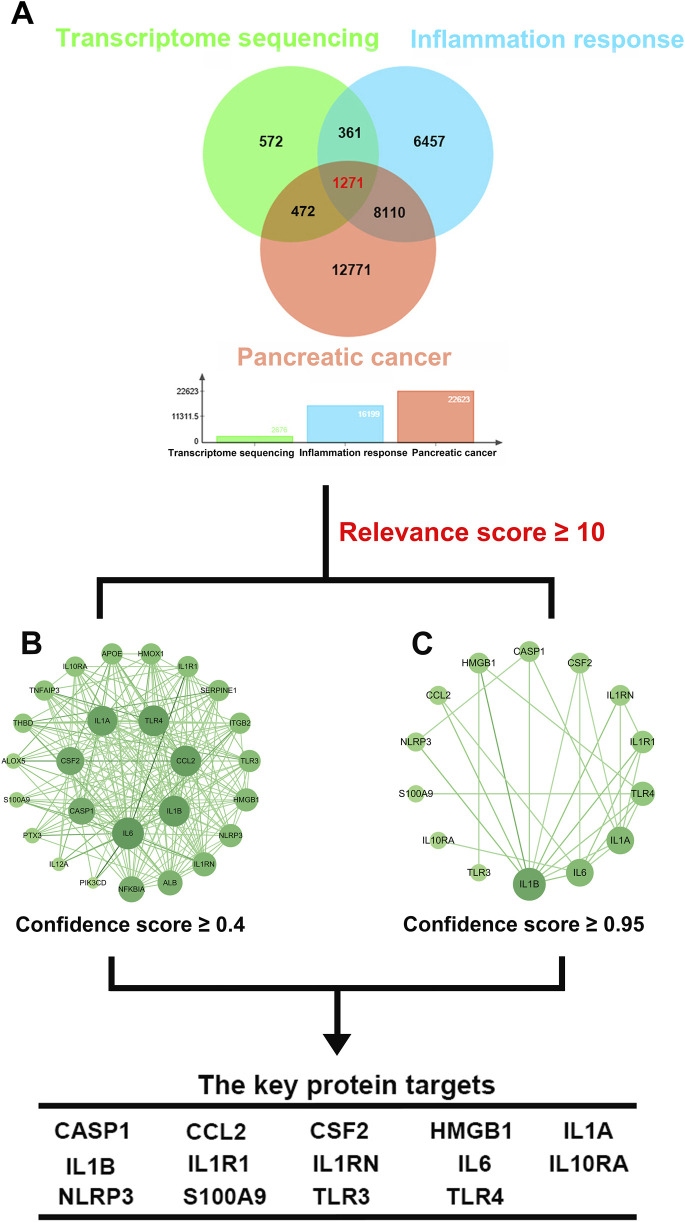
Intersection gene targets and Protein-protein interaction (PPI) network. **(A)** Intersection gene targets of transcriptome sequencing, pancreatic cancer and inflammatory response, **(B)** PPI network of protein targets, **(C)** PPI network of key protein targets (confidence >0.95).

This study focused on effect of curcumin on inflammatory response in pancreatic cancer. Therefore, we obtained 22,623 gene targets related to pancreatic cancer and 16,199 gene targets related to inflammatory response through GeneCards. Furthermore, we intersected the differentially expressed genes from transcriptome sequencing with genes related to pancreatic cancer and inflammatory response, resulting in 1,271 gene targets (Figure 3A). A GeneCards inflammation response-related score ≥10 was regarded as a key indicator for genes involved in regulation of inflammation by curcumin in pancreatic cancer, leading to identification of 27 gene targets. The PPI network was constructed using STRING, and by setting the confidence score threshold to 0.95, 14 key protein targets were identified, including IL1B, IL6, IL1A, TLR4, IL1R1, IL1RN, CSF2, CASP1, HMGB1, CCL2, NLRP3, S100A9, IL10RA and TLR3 (Figures 3B,C). Protein-Protein interaction analysis indicated that most proteins were involved in activation of inflammatory responses and recruitment of inflammatory cells, as well as the regulation of inflammatory responses and release of inflammatory factors.

### Processing of GSE28735 data and machine learning

**FIGURE 4:**
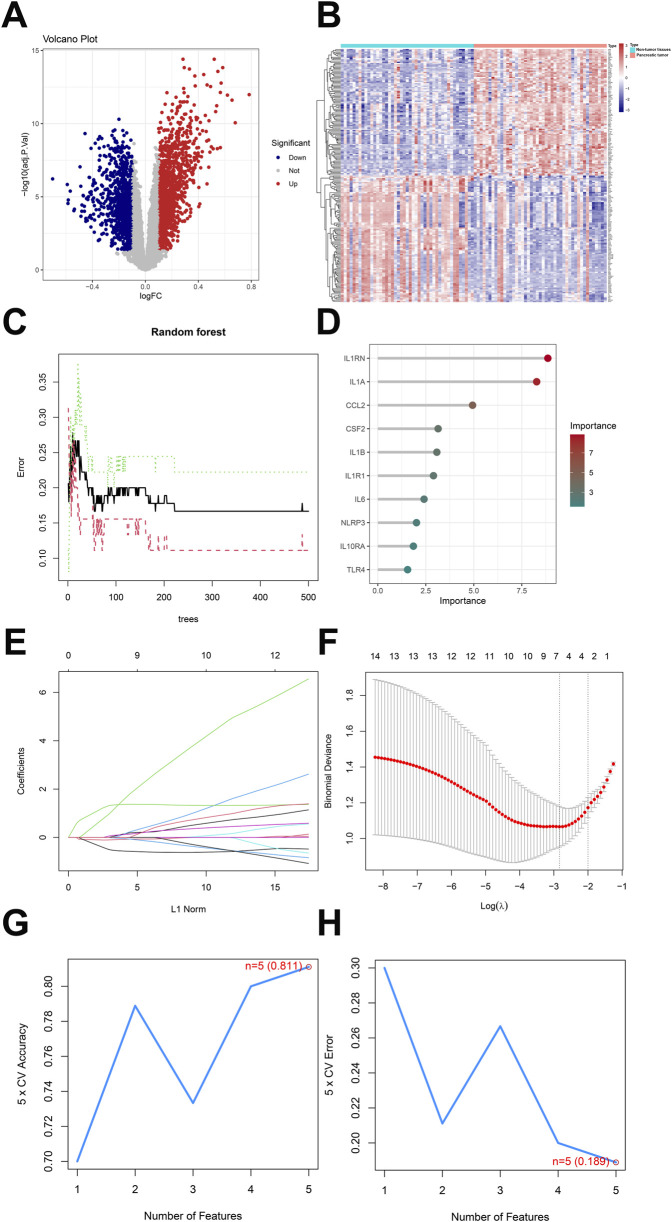
Analysis of GSE28735 and machine Learning for random forest, LASSO and SVM-RFE. **(A)** Volcano map of differentially expressed genes in GSE28735, **(B)** Heatmap of differentially expressed in GSE28735, **(C)** Random forest, black line indicated the training error, red line indicated the cross-validation error and green line indicated the standard deviation of the error, **(D)** Feature importance analysis, **(E)** LASSO regression coefficients plot, coefficients of different features in the LASSO regression model as a function of the number of L1 norms, **(F)** Binomial deviation of logistic regression, the binomial deviation of the logistic regression model as the logarithm λ varies, **(G)** The cross-validation accuracy of SVM-RFE, the best number of features was five and the accuracy was 0.811, **(H)** The cross-validation error of SVM-RFE, the cross-validation error has a best eigenvalue of five and an error of 0.189.

In GSE28735 data, there were 2,405 differentially expressed genes in tumors of pancreatic cancer patients compared to adjacent non-tumor tissues, with 1,028 genes having decreased expression and 1,377 genes having increased expression (Figures 4A,B). Three machine learning methods (RF, LASSO and SVM-RFE) were used to identify and analyze the association of differentially expressed genes in tumor tissues and adjacent non-tumor tissues of pancreatic cancer patients. Random Forest (RF) identifies the most important feature genes by evaluating the contribution of different genes to the classification model. As the number of trees increased, the error of the model gradually decreased, indicating accuracy and stability of the model. Notably, when number of trees reached 200 to 300, the error of the model stabilized, reflecting the strong adaptability of the random forest approach (Figure 4C). In the feature importance analysis, IL1RN, IL1A, CCL2, CSF2, IL1B, IL1R1, IL6, NLRP3, IL10RA and TLR4 showed a close association (Figure 4D). LASSO analysis selected the most predictive genes associated with pancreatic cancer by imposing L1 regularization. As the number of L1 paradigms increased, the coefficients of several genes gradually contracted to zero, and six key genes (CCL2, CSF2, IL1A, IL1B, IL1RN and IL6) were finally selected. This process not only helps to reduce the complexity of the model, but also improves the interpretability of the model (Figures 4E,F). SVM-RFE further optimized the model by recursively eliminating unimportant features. As the number of features increased, the accuracy of the model gradually improved, and the best accuracy reached 0.811 at a feature number of 5. Meanwhile the cross-validation error at different number of features, the optimal number of features was also 5, and the lowest error was 0.189. This result suggested that CSF2, IL1RN, IL6, HMGB1 and TLR4 selected by SVM-RFE were biologically important in the inflammatory response of pancreatic cancer (Figures 4G,H). In this study, key genes with critical regulatory roles in the inflammatory microenvironment of pancreatic cancer were systematically screened through cross-validation of multiple machine learning algorithms, providing a solid molecular foundation for the in-depth understanding of the inflammatory mechanisms in pancreatic cancer occurrence and progression.

### Survival analysis, pan-tumor analysis, immune infiltration analysis and single-cell transcriptome analysis

**FIGURE 5:**
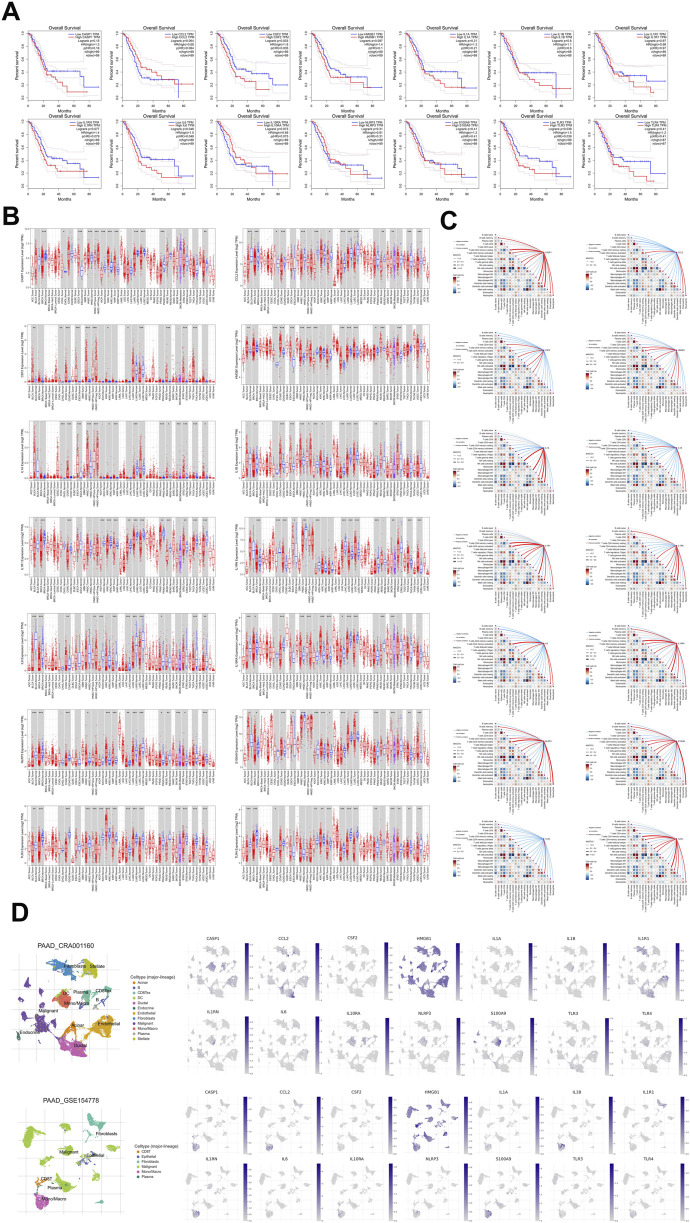
Expression and prognostic analysis of key differentially expressed genes. **(A)** Results of Survival analysis, **(B)** Results of pan-tumor analysis, **(C)** Results of immune infiltration analysis. **(D)** Result of single-cell transcriptome in CRA001160 and GSE154778.

We observed by survival analysis that low and high expression groups of different genes showed distinct trends in overall survival of pancreatic cancer patients. CSF2 (*p* = 0.033, HR = 1.16), IL6 (*p* = 0.048, HR = 1.5) and TLR3 (*p* = 0.039, HR = 1.5) showed statistically significant differences. Overall survival was significantly lower in patients with high expression of CSF2, IL6 or TLR3 compared to patients in low expression group. Results suggested that CSF2 may enhance tumor pro-inflammatory or pro-proliferative effects by stimulating myeloid cell proliferation and immune cell differentiation. This result also fit with the function played by IL6 in pro-tumor angiogenesis and immunosuppression-related mechanisms, and were consistent with the role played by TLR3 in driving inflammatory pathways and immune escape mechanisms. CCL2 (*p* = 0.064, HR = 0.68), IL1RN (*p* = 0.077, HR = 1.4) and IL10RA (*p* = 0.073, HR = 0.69) did not reach the traditional thresholds of significance, but the CCL2 chemokine function was critical for monocyte and macrophage recruitment and continues to warrant further attention. And the high expression of IL10RA may have a certain protective effect on the prognosis of patients, and IL10RA deserves to be studied in depth at the level of anti-inflammatory and negative regulation of immune response. CASP1 (*p* = 0.15, HR = 1.3), HMGB1 (*p* = 0.097, HR = 1.4), IL1A (*p* = 0.21, HR = 1.3), IL1B (*p* = 0.8, HR = 1.1), IL1R1 (*p* = 0.97, HR = 0.99), NLRP3 (*p* = 0.31, HR = 0.81), S100A9 (*p* = 0.41, HR = 1.2) TLR4 (*p* = 0.41, HR = 1.2) showed some trend but did not reach significant difference with survival prognosis (Figure 5A). In the pan-tumor analysis, CASP1 expression was slightly upregulated in most solid tumors relative to normal tissues, suggesting that the potential function of CASP1 associated with tumor inflammatory vesicle activation and apoptosis is prevalent. However, CASP1 expression was lower in lung tumors such as LUAD or LUSC relative to normal tissues, suggesting that the mechanism of CASP1 action may be limited by tissue specificity. CCL2 expression was lower in most tumors relative to normal tissues, which may stem from differences in the degree of immune infiltration of tumors such that monocyte and macrophage chemotaxis was not achieved to the same extent in all cancer types. CSF2 showed significant upregulation in a variety of tumors, suggesting that the function of CSF2 in stimulating differentiation and proliferation of bone marrow-derived cells were prevalent in tumor progression. HMGB1 expression was relatively uniformly elevated in most cancer types, which could be attributed to the broad and conserved role of HMGB1 as a “danger signal” in regulation of inflammatory activation, DNA repair and apoptosis in tumor cells. Both IL1B and IL1A demonstrated extensive and significant upregulation in pan-tumor analysis, and the results illustrate the critical roles played by IL1A and IL1B in reconstitution of local inflammation and distant immune networks in tumors, and in particular role of IL1B in inducing immune imbalance in tumor microenvironment as well as tumor cell infiltration and proliferation. IL1R1 and IL1RN expression varied widely in different tumors, suggesting that ligand-receptor-antagonist regulation may possess different equilibrium points in different tumor environments. Although the overall expression changes of IL6 and IL10RA were not as prominent as those of other inflammatory genes, the roles of IL6 and IL10RA in regulating inflammation and immune activation were still important in specific tissue settings. The expression changes of NLRP3 and S100A8 in various solid tumors varied widely, suggesting that NLRP3 and S100A8 had multiple regulatory functions in regulating inflammatory vesicle formation with tumor immune evolution and inflammatory pathways. Expression changes of TLR3 and TLR4 in different cancers were basically consistent, which was closely related to multiple roles of TLR3 and TLR4 as pattern recognition receptors in driving tumor-associated inflammatory cascades, activating immunity and even regulating metabolic reprogramming (Figure 5B). The results of immune infiltration analysis suggested that CASP1 was usually closely associated with the inflammatory vesicle pathway, and high CASP1 expression was often accompanied by an increase in M_2_ macrophages and dendritic cells, as well as a possible positive correlation with activated T cells CD4. CCL2, as a typical chemokine, was closely associated with neutrophils and eosinophils, while negatively regulating plasma cells. CSF2 was tightly linked to T cell regulatory (Tregs) and NK cells, and regulated antigen presentation, resulting in a complex phenomenon of parallel immunosuppression and immune activation. HMGB1 was significantly positively correlated with T cell gamma delta and macrophages M_2_, and was also associated with dendritic cell activation, as well as coupling with TLR4 signaling to trigger inflammatory amplification. Both IL1B and IL1A belong to IL-1 family, and IL1A and IL1B often act synergistically. IL1A was positively correlated with NK cells and dendritic cells, reflecting that IL1A could both enhance early immune response and promote immunosuppression through sustained inflammation. Meanwhile, the regulation of neutrophils by IL1B was also of interest. IL1R1 was positively correlated with regulating T cells CD4 and macrophages M_2_, implying that IL-1 signaling regulated tumor microenvironmental homeostasis and immune response through different immune cells. IL1RN was significantly positively correlated with dendritic cells, suggesting that when IL-1 pathway was antagonized, tumors may influence the inflammatory response by recruiting immunomodulatory cell populations. IL6 was significantly positively correlated with neutrophils and T cells CD4. IL10RA was positively correlated with macrophages M_2_ and T cells CD4, which was consistent with the mechanism that IL10RA negatively regulated inflammation and maintains immune homeostasis. NLRP3, a core component of classical inflammatory vesicles, was significantly positively correlated with macrophages M_2_, Neutrophils and activated T cells CD4, suggesting that NLRP3 activation promotes pro-inflammatory cellular responses. S100A9 was regarded as a myeloid-derived suppressor cell-associated protein, and S100A9 was strongly positively correlated with neutrophils. Meanwhile, S100A9 had some association with macrophages M2, suggesting that S100A9 actively promoted the intertwining of immune escape and inflammatory response in the tumor microenvironment. TLR3 was mainly positively associated with dendritic cells, while TLR4 was closely associated with macrophages M_2_ and T cells CD4 (Figure 5C). In single-cell transcriptome analysis of CRA001160 and GSE154778, CASP1 expression was significantly concentrated in the Mono-Macro (monocyte/macrophage) population, and there was almost no expression signal for CASP1 in other cell types. The enrichment of CASP1 in macrophages suggested a significant inflammatory response within the pancreatic cancer microenvironment, which may drive local immunosuppression and tumor progression. CCL2 and CSF2 were predominantly distributed in Mono-Macro cells, and cancer-associated fibroblast (CAF) showed sporadic expression. CCL2 was responsible for recruiting bone marrow-derived immunosuppressive cells and promoting the construction of a tumor immunosuppressive environment. CAF could further regulate the myeloid immune cell phenotype through the secretion of CSF2, creating a pro-inflammatory and immunosuppressive stromal environment. HMGB1 expression was dominated by Malignant (malignant cells). And HMGB1 was distributed in a variety of cells, but tumor cells showed stronger expression. The high expression of HMGB1 in malignant cells reflected the fact that cancer cells were continuously subjected to stress or death-driven activation of the intrinsic immune system and maintenance of a chronic inflammatory microenvironment. IL1A and IL1B were the dominant expression populations in Mono-Macro cells and weakly expressed in other cell types. The distribution pattern of IL1RN and IL1R1 was close to each other, and the monocyte/macrophage population was the main expression area of IL1RN and IL1R1. IL6 had significant signals in both Mono-Macro and CAF and was weakly expressed in some malignant cells. IL10RA was mainly concentrated in CD8Tex and Mono-Macro. NLRP3 expression was extremely restricted to Mono-Macro population, and the signaling distribution of NLRP3 was very concentrated. S100A9, TLR3 and TLR4 expression was also predominantly seen in Mono-Macro, with sparse expression in non-immune cell compartments. Although the overall cell numbers and distributions of CRA001160 and GSE154778 were slightly different, the expression patterns, signal-enriched regions and expression comparisons among different cell types of all genes were very consistent, reflecting the high reproducibility and stability of the results. The results indicated that most inflammation and immunity-related genes were highly enriched in monocytes/macrophages, and some fibroblasts could also express specific factors. Malignant tumor cells showed limited expression of all genes in tumor cells, except for HMGB1, which had prominent high expression. Single-cell transcriptome analysis revealed that active expression of specific inflammatory and immune-regulated genes in tumor microenvironment was concentrated in immune and stromal cells and was limited in malignant cells (Figure 5D).

### Differential expression of key targets

**FIGURE 6:**
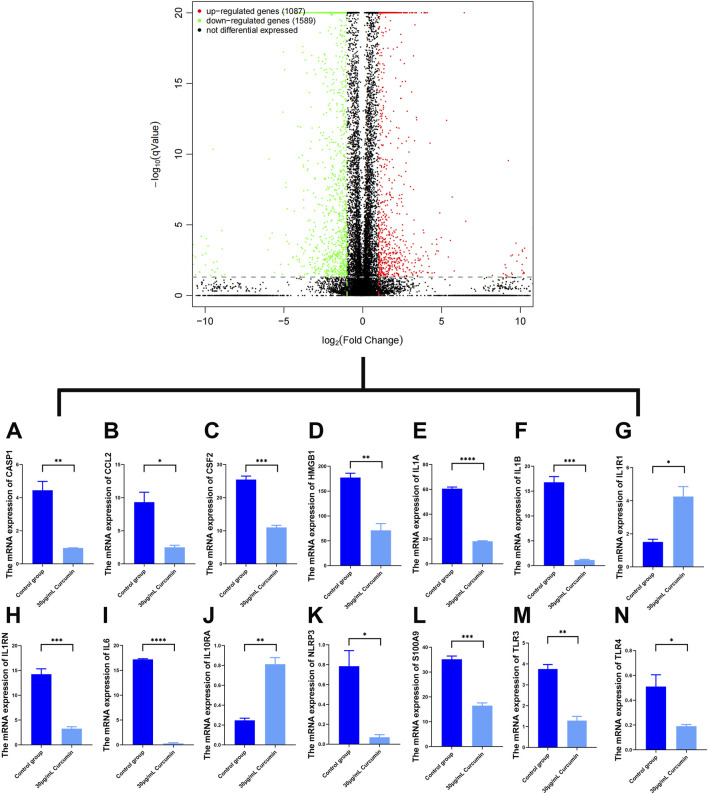
Expression of the key differential target mRNA (n = 3). **(A)** The mRNA expression of CASP1, **(B)** The mRNA expression of CCL2, **(C)** The mRNA expression of CSF2, **(D)** The mRNA expression of HMGB1, **(E)** The mRNA expression of IL1A, **(F)** The mRNA expression of IL1B, **(G)** The mRNA expression of IL1R1, **(H)** The mRNA expression of IL1RN, **(I)** The mRNA expression of IL6, **(J)** The mRNA expression of IL10RA, **(K)** The mRNA expression of NLRP3, **(L)** The mRNA expression of S100A9, **(M)** The mRNA expression of TLR3, **(N)** The mRNA expression of TLR4.

As we detected changes in mRNA expression in both the control group and 30 μg/mL curcumin-treated group through transcriptome sequencing, we compiled the expression changes of 14 key targets mRNA previously identified. Transcriptome sequencing results indicated that the expressions of mRNA for CASP1, CCL2, CSF2, HMGB1, IL1A, IL1B, IL1RN, IL6, NLRP3, S100A9, TLR3 and TLR4 were significantly inhibited following curcumin treatment (Figures 6A–F,H,I, K, L, M, N). These targets each played critical roles in inflammatory response. Activation of NLRP3, TLR3 and TLR4 may lead to chronic inflammation, which contributed to immune evasion and tumor progression. CCL2 counteracted the anti-tumor immune response by recruiting immunosuppressive cells, such as regulatory T-cells and myeloid-derived suppressor cells, to the tumor site. HMGB1 and S100A9 were involved in amplifying signals of cell damage and inflammation, and they could also promote cell migration and invasion within tumors. CASP1 played a role in formation of inflammasomes and activation of pro-inflammatory cytokines; it may influence immune responses and tumor cell death by regulating pyroptosis and inflammasome activity. Meanwhile, curcumin treatment upregulated mRNA expression of IL10RA and IL1R1 (Figures 6G,J). The upregulation of IL10RA could enhance anti-inflammatory effect of IL-10, protecting normal tissues by preventing tissue damage caused by over-inflammation and inhibiting excessive inflammatory response. Curcumin inhibited pro-inflammatory and immunosuppression-related gene expression, and upregulated anti-inflammatory and immunomodulatory genes through multi-targets, providing a new molecular basis for blocking tumor-associated chronic inflammation and improving the immune microenvironment.

### Molecular docking results

**FIGURE 7:**
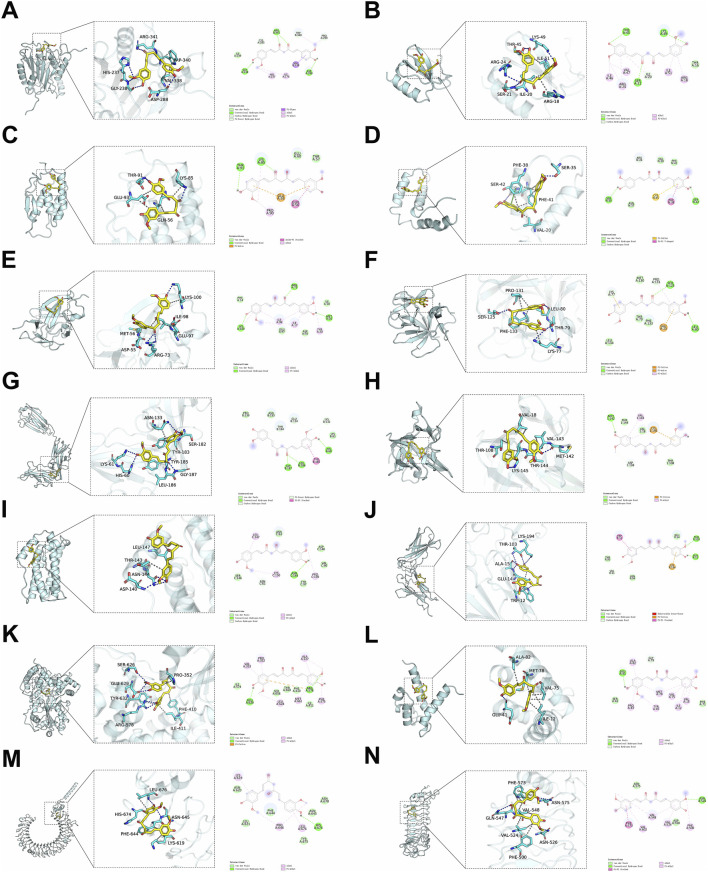
Molecular docking of curcumin and protein targets. **(A)** CASP1/Curcumin, **(B)** CCL2/Curcumin, **(C)** CSF2/Curcumin, **(D)** HMGB1/Curcumin, **(E)** IL1A/Curcumin, **(F)** IL1B/Curcumin, **(G)** IL1R1/Curcumin, **(H)** IL1RN/Curcumin, **(I)** IL6/Curcumin, **(J)** IL10RA/Curcumin, **(K)** NLRP3/Curcumin, **(L)** S100A9/Curcumin, **(M)** TLR3/Curcumin, **(N)** TLR4/Curcumin.

Curcumin contained two coumarin rings and an α, β-unsaturated ketone structure. The α, β-unsaturated ketone structure of curcumin provided a reactive site that could form covalent bonds with specific amino acid groups and also exerts regulatory effects by altering the activity of downstream signaling molecules. The hydroxyl groups in the coumarin rings of curcumin could form hydrogen bonds with amino acid residues in proteins including glycine, glutamic acid and lysine. These hydrogen bonds facilitated the specific binding of curcumin to proteins. The arrangement of hydrogen bond acceptors and donors in amino acids could also influence the affinity of the binding. Furthermore, the phenolic structure of curcumin enabled it to interact with various non-polar amino acids through hydrophobic interactions. The combination of electrostatic interactions, hydrophobic interactions and hydrogen bonds collectively provided a synergistic effect that facilitates the binding of curcumin to its target protein. This effect also aided curcumin in locating ideal binding pockets within structurally complex proteins. Curcumin interacted with CASP1 at multiple key amino acid sites, namely, Trp-340, Pro-290, Cys-285, His-237 and Ile-176, forming hydrophobic interactions, while Gly-238, Arg-341, and Asp-288 were involved in hydrophilic contacts (Figure 7A). With CCL2, curcumin established hydrophobic interactions at Ile-46, Val-47, Arg-24, Ile-20, Ile-51 and Arg-18, highlighted by π-alkyl interactions with Arg-24, Ile-20, Ile-51 and Arg-18, and hydrophilic interactions at Thr-45, Lys-49 and Ser-21 (Figure 7B). Additionally, curcumin engaged with CSF2 through hydrophilic interactions involving Thr-91, Lys-85 and Glu-93, including a pi-anion bond with Glu-93 that enhanced stability, alongside hydrophobic interactions at Pro-90 and Gln-56 (Figure 7C). Curcumin interacted hydrophobically with Arg-24, Cys-45 and Phe-38 on HMGB1. Notably, curcumin established pi-Sulfur interactions with Cys-45, and it established pi-pi T-shaped interactions with Phe-38. Beyond its hydrophobic interactions, curcumin displayed hydrophilic contacts with Ser-35 and Ser-42, reinforcing its binding to HMGB1 (Figure 7D). For IL1A, curcumin engaged in hydrophobic interactions at Lys-100, Ile-98, Ile-17 and Tyr-20, alongside hydrophilic interactions with Lys-100, Arg-73 and Met-56 (Figure 7E). Meanwhile, for IL1B, curcumin established hydrophilic contacts with Glu-25, Lys-77, Ser-125 and Leu-80, while also exhibiting hydrophobic interactions at Pro-131 and Lys-77 (Figure 7F). Curcumin interacted hydrophobically with Thr-184, Glu-134, Lys-132 and Tyr-185 on IL1R1. Notably, curcumin established pi-pi stacked interactions with Tyr-185. In addition to its hydrophobic interactions, curcumin also established hydrophilic contacts with Gly-187, Leu-186 and His-60 on IL1R1 (Figure 7G). On IL1RN, it formed hydrophilic interactions with Met-142 and Lys-145, including a π-cation bond with Lys-145. Curcumin also displayed hydrophobic contacts with Asp-138 and Val-143 (Figure 7H). Regarding IL6, curcumin engaged in hydrophobic interactions with Leu-47, Asn-61, Lys-150 and Pro-139, as well as a π-alkyl interaction with Leu-147, while forming a hydrophilic bond with Asn-144 (Figure 7I). Curcumin interacted hydrophobic interactions with Trp-12 and Val-11 on IL10RA. This hydrophobic interaction allowed curcumin to better embed itself into the pocket structure of the receptor protein, thereby affecting the conformation and function of IL10RA. It was noteworthy that curcumin established pi-pi stacked interactions with Trp-12. Furthermore, curcumin interacted hydrophilically with IL10RA through Lys-194, Thr-103 and Ala-15, and it established pi-cation interactions with Lys-194 (Figure 7J). Curcumin formed hydrophilic interactions with several residues on NLRP3, notably Glu-629 and Arg-578, along with a π-cation interaction with Arg-578. In addition, it exhibited hydrophobic interactions with Val-353, Pro-352, Leu-628, Ala-227, Phe-575 and Met-661, and established π-alkyl interactions with Val-353, Pro-352, Ala-227 and Met-661 (Figure 7K). With S100A9, curcumin engaged in hydrophobic interactions at residues Ala-82, Pro-43, Tyr-45, Met-78, Val-75, Ile-12 and Val-15, while also demonstrating hydrophilic interactions with Glu-41 (Figure 7L). Curcumin interacted hydrophobic interactions with Lys-619, Pro-646 and Leu-676 on TLR3, and it established π-alkyl interaction with Lys-619 and Leu-676. These stable hydrophobic interactions helped curcumin to embed itself in the binding pocket of TLR3, thereby inhibiting subsequent inflammatory signalling. Furthermore, curcumin interacted hydrophilically with TLR3 through His-674 (Figure 7M). Curcumin interacted hydrophobic interactions with Val-602, Phe-573, Val-548, Val-524 and Phe-500 on TLR4. Notably, curcumin established pi-pi stacked interactions with Phe-573. Alongside its hydrophobic interactions, curcumin also exhibited hydrophilic interactions with Asn-526 (Figure 7N).

### Molecular dynamics results

**FIGURE 8:**
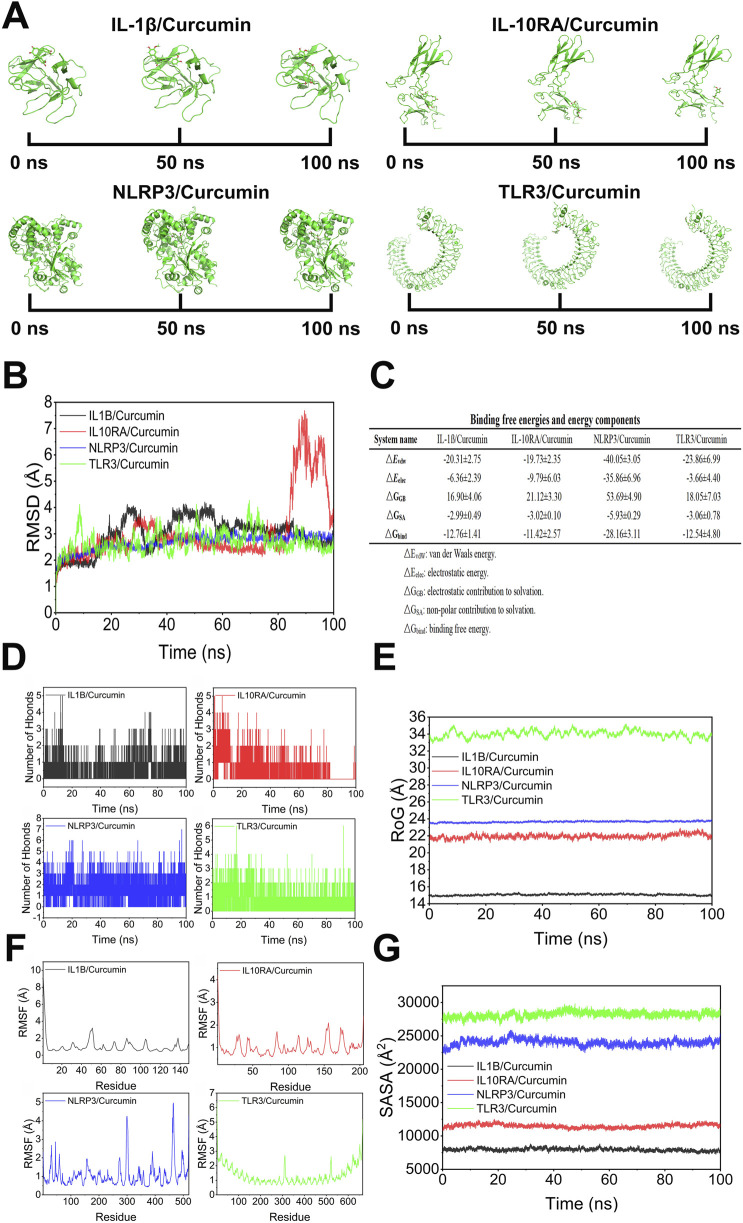
The result of molecular dynamics. **(A)** Binding states of curcumin and protein in molecular dynamics simulations, **(B)** Root mean square deviation (RMSD), **(C)** Binding free energies and energy components predicted by MM/GBSA (kcal/mol), **(D)** Hydrogen bonding, **(E)** Radius of gyration (RoG), **(F)** Root mean square fluctuations (RMSF), **(G)** Solvent accessible surface area (SASA).

In the results of molecular dynamics, RMSD fluctuations of NLRP3/Curcumin were extremely stable, indicating that the binding between curcumin and NLRP3 was very stable. The stable binding of NLRP3/Curcumin relied on the appropriate interactions between the spatial configuration of curcumin and the binding site of NLRP3. The stability of the RMSD suggested that curcumin could maintain an ideal structure and spatial arrangement within the binding pocket of NLRP3, which may be related to hydrophobic nature of curcumin and its strong hydrophobic interactions with the non-polar amino acid residues inside NLRP3, allowing for a stable binding state of NLRP3/Curcumin. The binding of IL1B/Curcumin showed convergence in later simulations, achieving moderate stability. The moderate stability of curcumin with IL1B may suggest its flexibility in modulating this inflammatory factor. Interestingly, the structural characteristics of IL1B made it more adaptable to small molecule drugs, and the binding of curcumin may be achieved through multiple modes of action, meaning that after binding, IL1B undergoes a certain conformational adjustment to accommodate curcumin. The RMSD of TLR3 showed considerable fluctuation with moderate to low stability, indicating role of curcumin in modulating TLR3 pathway may be relatively limited. Curcumin’s effect on TLR3 might require a stronger binding ability, the current interaction could only partially interfere with TLR3 signaling, rather than completely blocking the inflammatory response triggered by TLR3. Additionally, the binding pocket of TLR3 was large, and curcumin may not be able to fully embed deeply within it, resulting in its binding stability being less than that of IL1B and NLRP3. Binding of IL10RA showed significant fluctuations in the later stages of the simulation, suggesting that curcumin may bind to this protein in a relatively unstable manner. In the early stages, curcumin likely formed certain interactions with IL10RA due to its unique chemical groups, but the forces of interaction after binding were insufficient to maintain a high level of stability. This result indicated that the effectiveness of curcumin in regulating IL-10 pathway and inhibiting inflammatory responses may be limited (Figures 8A,B). The binding free energies for IL1B/Curcumin, IL10RA/Curcumin, NLRP3/Curcumin and TLR3/Curcumin were −12.76 ± 1.41 kcal/mol, −11.42 ± 2.57 kcal/mol, −28.16 ± 3.11 kcal/mol and −12.54 ± 4.80 kcal/mol, respectively. The binding free energy for NLRP3/Curcumin, which was the highest at −28.16 ± 3.11 kcal/mol, further confirmed the strength and stability of the binding between NLRP3 and curcumin. This result suggested that in the biological context of regulating the inflammatory response, the binding of curcumin to NLRP3 was more pronounced than with other proteins, and the activation of NLRP3 was directly related to the release of pro-inflammatory factors. Lower binding free energies suggested binding stability of IL1B/Curcumin, IL10RA/Curcumin and TLR3/Curcumin were relatively weak, which may limit their roles in regulation of inflammatory responses (Figure 8C). The number of hydrogen bonds formed in NLRP3/Curcumin was high and stable, demonstrating the superiority of their binding. In contrast, the number of hydrogen bonds of IL1B/Curcumin, IL10RA/Curcumin and TLR3/Curcumin in later stages of this simulation significantly decreased. Notably, this sharp decline in the number of hydrogen bonds in IL10RA/Curcumin indicated contribution of hydrogen bonds to the subsequent binding of IL10RA/Curcumin gradually weakened. The changes in the number of hydrogen bonds could not only explain the variations in binding but also reveal the self-regulation that curcumin experiences with these proteins during the binding process (Figure 8D). The radius of gyration (RoG) of NLRP3/Curcumin and IL1B/Curcumin remained within a small range of fluctuations, indicating a high binding affinity and compact structure. In contrast, IL10RA/Curcumin and TLR3/Curcumin exhibited greater variability in RoG, suggesting a looser structure and potentially weaker binding (Figure 8E). In the results of RMSF, all proteins that bound with curcumin exhibited an overall reduction in flexibility, especially in the core regions where the RMSF values were generally below 2.5 Å. This phenomenon indicated that the binding of curcumin enhanced the conformational stability of these proteins, aligning with the expectations that small molecule drugs stabilize target proteins. In the inflammatory response, curcumin could weaken the flexibility and reactivity of inflammation-related target proteins through its binding, thereby modulating the inflammatory response. Interestingly, NLRP3, IL1B and TLR3 exhibited relatively strong rigidity after binding with curcumin, suggesting that this interaction has a significant inhibitory effect on their activities (Figure 8F). In solvent accessible surface area (SASA) analysis, the fluctuations of NLRP3/Curcumin, IL1B/Curcumin, IL10RA/Curcumin and TLR3/Curcumin were stable, indicating that the changes in the surface exposure and buried regions of each complex were relatively small (Figure 8G).

## Discussion

This study found curcumin downregulated TLR3 expression and interfered with NLRP3 activation to block the release of inflammatory vesicles and NF-κB signalling pathway, thereby directly interfering with IL1B activation. In addition, curcumin upregulated IL10RA expression to promote IL-10 activation and JAK/STAT signalling pathway, thereby interfering with IL1B and its downstream signalling pathway.

IL1B, as a pro-inflammatory cytokine, played an essential role in chronic inflammation and tumor growth (Rébé and Ghiringhelli, 2020). Release and production of IL1B are regulated by various upstream signals including NLRP3 inflammasome (Malkova et al., 2023; He et al., 2016). Activation and assembly of NLRP3 inflammasome lead to activation of CASP1 (Chen et al., 2021a). And CASP1 further catalyzes the cleavage of pro-IL1B, resulting in generation of IL1B, which is released extracellularly (Zangiabadi and Abdul-Sater, 2022). IL1B played multiple roles in inflammatory response (Jo et al., 2016). IL1B not only promoted activation and recruitment of inflammatory cells but also enhanced the production of pro-inflammatory factors including TNF-α and IL6, thereby creating a positive feedback loop that further exacerbated inflammatory response (Tylutka et al., 2024; Raziyeva et al., 2021; Lopez-Castejon and Brough, 2011). IL1B activates downstream pathways including MAPK and NF-κB through binding to IL-1R, leading to the persistence and enhancement of inflammatory response (Ahmed et al., 2023; Pfeiler et al., 2019; Pang et al., 2023). Research found that IL1B not only promoted proliferation and survival of pancreatic cancer but also enhanced tumor metastasis and invasion by influencing polarization of immune cells within tumor microenvironment (Siddiqui et al., 2017; Herremans et al., 2022; Dosch et al., 2021). Some studies indicated high IL1B expression was associated with poor prognosis in patients with pancreatic cancer (Oberstein et al., 2024; Das et al., 2020). Besides, curcumin can significantly inhibit LPS-induced NF-κB activation, thereby reducing NLRP3 expression (Lan et al., 2023; Kandikattu et al., 2020; McKay et al., 2008). Therefore, curcumin reduces tissue damage and chronic inflammation by inhibiting activity of IL1B, thereby affecting tumor microenvironment. And curcumin suppresses tumor migration and proliferation by interfering with IL1B and its downstream pathways.

NLRP3, as a component of inflammasome, plays a key role in inflammatory responses and immune system (Ma, 2023). Assembly of inflammasome and activation of NLRP3 are initiated by sensing pathological stimuli both inside and outside the cell, including pathogen infections, cellular damage and endogenous danger signals (Xia et al., 2023). Activation of NLRP3 is generally divided into two stages: the priming stage and the activation stage (Kelley et al., 2019). In the initial signaling phase, bacterial toxins, viral infections or inflammatory factors trigger NF-κB pathway, leading to upregulation of NLRP3 transcription and synthesis of its precursor proteins (Fu and Wu, 2023). During this process, NLRP3, as a pattern recognition receptor, continuously increases its expression while preparing the conditions for the subsequent activation stage (Mangan et al., 2018). During activation phase, NLRP3 induces self-assembly by recognizing metabolic products (including: ATP or intracellular ROS), NLRP3 forms a complex with CASP1 and ASC to create NLRP3 inflammasome (Huang et al., 2021; Shao et al., 2015). The abnormal activation of NLRP3 is strongly linked to several chronic inflammatory diseases, such as metabolic syndrome, rheumatoid arthritis, and pancreatic cancer (Deng et al., 2022; Jin et al., 2023). In pancreatic cancer, NLRP3 and its mediated inflammatory response are considered to significantly impact progression and occurrence of tumor (Lutz et al., 2023; Boone et al., 2019; Zheng and Liu, 2022). Pancreatic cancer often produced large amounts of ROS due to metabolic abnormalities. These ROS not only directly activated NLRP3 inflammasome but also further promoted inflammatory response by inducing cell apoptosis, necrosis and releasing endogenous danger signals, thus creating a vicious cycle (Gao et al., 2015; Zhong et al., 2022). NLRP3 can affect immune microenvironment of pancreatic cancer by regulating polarization state of tumor-associated macrophages. Activation of NLRP3 may enhance M_2_ polarization of tumor-associated macrophages, thereby promoting tumor metastasis and growth (Amo-Aparicio et al., 2023). A study found inhibiting the activity of NLRP3 inflammasome may have anti-tumor effects (Wang et al., 2016). Research has found that tumor growth is significantly slowed by the use of NLRP3 antagonists or gene knockout techniques, accompanied by a substantial decrease in inflammatory factors (Tezcan et al., 2021; Xue et al., 2019). Studies found curcumin reduced NLRP3 expression by inhibiting NF-κB activation, thereby interfering with inflammatory responses and tissue damage (Ran et al., 2021; Kong et al., 2016; Tomeh et al., 2019). And curcumin reduced NLRP3 activation by inhibiting upstream regulatory factors of NLRP3 including MAPK pathway (Shamsnia et al., 2023; Wang and He, 2022). Curcumin weakened stimulation of NLRP3 induction factors (including: ROS and potassium ion efflux) by scavenging free radicals and regulating intracellular potassium ion concentration (Chen et al., 2024; Barchitta et al., 2019). Therefore, considering role of NLRP3 in tumor microenvironment, on one hand, curcumin can effectively reduce the pro-inflammatory microenvironment of pancreatic cancer by regulating activity of NLRP3 inflammasome, thereby inhibiting tumor progression; on the other hand, activation of NLRP3 may be associated with tumor resistance.

IL10RA is an important component of IL-10 signaling (Wang et al., 2019). IL-10 is a powerful anti-inflammatory factor produced by immune cells including T cell, B cell and macrophage (Saraiva and O'Garra, 2010; Moore et al., 2001). IL-10 activates downstream signals like JAK by forming IL-10/IL10RA complex through its binding with IL10RA, subsequently activating transcription factors including STAT3 (Baldini et al., 2021; Salkeni and Naing, 2023). Phosphorylated STAT3 translocates to cell nucleus and participates in regulation of gene expression related to cell survival, proliferation and immune evasion (Henrik Heiland et al., 2019; Ouyang and O’Garra, 2019). IL-10 reduces expression of those pro-inflammatory factors by inhibiting activation of NF-κB and MAPK signaling pathways (Fang et al., 2022; Ouyang et al., 2011). The anti-inflammatory effect of IL-10 is achieved by inhibiting NF-κB pathway (Ziegler et al., 2021; Geginat et al., 2016). The suppression of NF-κB could reduce production of pro-inflammatory cytokine, thereby affecting tumor microenvironment (Toker et al., 2017). IL-10 can inhibit tumor metastasis and growth by modulating chronic inflammation in tumor microenvironment (Gorosito Serrán et al., 2015). Study indicated curcumin could enhance the anti-inflammatory response by upregulating IL-10 expression and strengthening signaling of IL10RA (Ge et al., 2023). Additionally, IL-10 may inhibit invasion and migration capabilities of tumor by modulating extracellular matrix components within tumor microenvironment (Hatab et al., 2019; Jalilian et al., 2023). Therefore, curcumin may play a critical role in development and occurrence of pancreatic cancer by regulating chronic inflammation in tumor microenvironment through modulation of IL10RA signaling.

TLR3, as an important pattern recognition receptor, plays a crucial role in immune system by monitoring viral infections and responding to pathological states (Chen et al., 2021b). TLR3, located inside the cell, was responsible for recognizing intracellular dsRNA, which was typically a hallmark of viral infection (Matsumoto and Seya, 2008; Nicodemus and Berek et al. (2010). Upon recognizing specific ligands, TLR3 initiates downstream signaling pathways by interacting with adaptor protein TRIF, thereby activating transcription factors including IRF3 and NF-κB (Schröder and Bowie, 2005; Takeda and Akira, 2004; Chattopadhyay and Sen, 2014). This process is not only significant in antiviral responses but also plays an important role in development of chronic inflammation and tumor (Wang et al., 2020; Kawai and Akira, 2008). In the TLR3 signaling pathway, dsRNA binds to TLR3, leading to a conformational change in TLR3 and the activation of the associated adaptor molecule TRIF. The activation of TRIF promotes IKK activation, which in turn activates NF-κB pathway (Seya et al., 2022; Grootjans et al., 2017). Activated NF-κB transcription factors translocate to nucleus and induce transcription of pro-inflammatory factors including IL6, IL-12 and TNF-α (Hu et al., 2024; Hoebe and Beutler, 2004). Activated TRIF can also activate IRF3 through another pathway, further regulating the production of interferons (Wang H. et al., 2023; Fujisawa et al., 2020; Yum et al., 2021). Study found overactivation of TLR3 led to prolonged pro-inflammatory responses, promoting formation of tumor microenvironment (Zou et al., 2022; Vaz and Andersson, 2014). Specifically, pancreatic cancer is a typical tumor closely associated with chronic inflammation, and there is a significant correlation between long-term pancreatitis and occurrence of pancreatic cancer (Hamada et al., 2014; Whitcomb, 2004; Castaño-Rodríguez et al., 2014; Marstrand-Daucé et al., 2023). Research indicated that curcumin could reduce production of pro-inflammatory cytokines through inhibiting TLR3-related signaling pathways, thereby alleviating local chronic inflammation (Boozari et al., 2019). Additionally, some studies found curcumin could modulate inflammatory responses and tumor microenvironment by decreasing the expression of TLR3 (Zhao et al., 2011; Lopes et al., 2016). IL-10 could inhibit excessive activation of TLR3, and the mechanism of IL-10 involves downregulation of NF-κB pathway, which alleviated inflammatory response in tumor microenvironment (Kanmani and Kim, 2019; Hu et al., 2016; Zhao et al., 2023). TLR3 may play a promoting role in the early stages of pancreatic cancer development by inducing inflammatory responses that activate secretion of pro-inflammatory factors, thereby stimulating growth of tumor cells (Gandhi et al., 2023; Kim et al., 2023). Therefore, during the progression of pancreatic cancer, TLR3 acts as a double-edged sword, serving as part of the immune system’s defense while potentially accelerating the malignant transformation of tumors due to chronic inflammation. Curcumin plays a significant role in inhibiting chronic inflammation and pro-inflammatory factors associated with pancreatic cancer by regulating TLR3 and its downstream signaling pathways.

## Conclusion

This research findings indicated that curcumin not only directly interfered with the activation of IL1B by blocking the activation of NLRP3 by TLR3, but also upregulated IL10RA expression to activate IL-10, thereby interfering with IL1B and its downstream signalling pathway.

The mechanism of curcumin involved downregulation of TLR3 expression and interference with NLRP3 activation to block the release of inflammatory vesicles and NF-κB signalling pathway, thereby directly interfering with IL1B activation. In addition, curcumin upregulated IL10RA expression to promote IL-10 activation and JAK/STAT signalling pathway, thereby interfering with IL1B and its downstream signalling pathway. Ultimately curcumin treats pancreatic cancer by interfering with IL1B to inhibit inflammatory response through modulation of multiple signalling pathways. Studies of curcumin’s pharmacological effects to inhibit pancreatic cancer growth by modulating inflammatory responses and inhibiting pancreatic cancer growth may offer new possibilities for pancreatic cancer treatment.

## Data Availability

The datasets presented in this study can be found in online repositories. The names of the repository/repositories and accession number(s) can be found in the article/supplementary material.

## Glossary

BP: Biological Process
CCL2: C- C Motif Chemokine Ligand 2
CASP1: Caspase 1
CC: Cell Component
CCK-8: Cell Counting Kit-8
CSF2: Colony Stimulating Factor 2
DMSO: Dimethyl sulfoxide
GO: Gene Ontology
HMGB1: High Mobility Group Box 1
IRF3: Interferon Regulatory Factor 3
IL1RN: Interleukin 1 Receptor Antagonist
IL1R1: Interleukin 1 Receptor Type 1
IL10RA: Interleukin 10 Receptor Subunit Alpha
IL1A: Interleukin-1 Alpha
IL1B: Interleukin-1 Beta
IL10: Interleukin-10
IL6: Interleukin-6
IL8: Interleukin-8
JAK: Janus Kinase
KEGG: Kyoto Encyclopedia of Genes and Genomes
LASSO: Least Absolute Shrinkage and Selection Operator
MTOR: Mechanistic Target Of Rapamycin Kinase
COX-2: Mitochondrially Encoded Cytochrome C Oxidase II
MAPK: Mitogen-Activated Protein Kinase
MF: Molecular Function
NLRP3: NOD-like receptor thermal protein domain associated protein 3
NF-κB: Nuclear Factor Kappa B
PGE2: Prostaglandin E2
PPI: Protein-Protein Interaction
RoG: Radius of Gyration
RF: Random Forest
ROS: Reactive Oxygen Species
RMSF: Root Mean Square Fluctuation
RMSD: Root Mean Square Deviation
S100A9: S100 Calcium Binding Protein A9
STAT3: Signal Transducer And Activator Of Transcription 3
SASA: Solvent Accessible Surface Area
SVM-RFE: Support Vector Machine Recursive Feature Elimination
TLR3: Toll Like Receptor 3
TLR4: Toll Like Receptor 4
TGF-β: Transforming Growth Factor-β
TIMER: Tumor Immune Estimation Resource
TNF-α: Tumor Necrosis Factor-α
